# Acute Practitioners’ Experiences of Implementing Frailty Same Day Emergency Care: A Researcher-in-Residence Study

**DOI:** 10.5334/ijic.9854

**Published:** 2026-06-18

**Authors:** Kieran Green, Sheena Asthana, John Downey, Joanne Watson

**Affiliations:** 1 Centre for Health Technology, University of Plymouth, Plymouth, UK; 2 Torbay and South Devon NHS Foundation Trust, Torquay, UK

**Keywords:** triage, digital integration, implementation, acute frailty care, ambulatory care

## Abstract

**Introduction:**

NHS policy promotes Frailty Same Day Emergency Care (F-SDEC) units to alleviate pressure on emergency departments (ED) and improve care for frail people through comprehensive geriatric assessment and same-day discharge. However, evidence remains limited on how such services are implemented in practice, particularly in resource-constrained and geographically peripheral settings. This study aimed to examine how implementation barriers are experienced and negotiated during the day-to-day delivery of an F-SDEC pilot.

**Methods:**

Using a Researcher-in-Residence approach, a researcher embedded in a coastal hospital in Torbay, South-West England, collected field notes from prior observations to inform the interview guide, conducted semi-structured interviews with staff delivering F-SDEC, and held a feedback session. A framework-informed, reflexive thematic approach guided the identification of key implementation challenges and enablers.

**Results:**

Staff viewed F-SDEC as beneficial for improving care for older adults, but interacting barriers constrained implementation. ED-based triage was time-intensive and difficult to routinise due to fragmented, non-interoperable IT systems, inconsistent understanding of frailty across hospital teams, and shifting eligibility criteria. Operational pressures, workforce and skill-mix shortages, and an inconsistent understanding of F-SDEC’s needs and purpose also limited CGA delivery and contributed to day-to-day variability in throughput. While staff expressed a desire to reorient towards community-based referrals, the same digital and workforce constraints also limited the feasibility of alternative routes, further raising sustainability concerns.

**Conclusions:**

F-SDEC implementation was shaped by interdependent system constraints rather than isolated barriers. In peripheral and resource-constrained settings, successful implementation may depend on whole-system alignment across digital infrastructure, workforce capacity, and cross-team collaboration, with national policy better accounting for local delivery conditions.

## Introduction

Frailty, defined as an accumulation of ‘deficits’ such as hearing loss or cognitive decline [1], is strongly associated with increased use of health and social care services [2,3]. This includes high rates of unscheduled urgent frailty care, which is increasing pressure on emergency departments (ED), inpatient beds, and producing poorer outcomes for older frail patients [4,5,6]. A significant proportion of ED visits are considered either inappropriate or avoidable [7,8].

Like other higher-income countries, the United Kingdom (UK) is already experiencing a significant prevalence of frailty. In England, between 2006 and 2017, recorded frailty prevalence in adults aged 50+ increased from 26.5% to 38.9%, and is expected to grow further [9]. In 2021/22, almost half of all people arriving in the ED by ambulance were over 65, with a third over 75 [10].

### Frailty Same Day Emergency Care (F-SDEC)

To ensure the provision of good-quality care and a sustainable health system, NHS England’s Long-Term Plan and FRAIL strategy state the need to implement anticipatory and preventive care models that help delay frailty and, when patients present to the emergency department (ED), prevent inappropriate hospitalisation and re-attendance [11]. The F-SDEC represents one such model. Patients are identified in the ED or Acute Medical Units (AMUs) and referred to the F-SDEC multidisciplinary team (MDT) for a rapid, comprehensive geriatric assessment (CGA) [12], diagnostic imaging, and same-day discharge for patients deemed capable of returning to their usual place of residence. Teams typically comprise a geriatrician, advanced nurse practitioners, physiotherapists and occupational therapists, nurses, and healthcare assistants [13,14]. However, there is no single established configuration for F-SDEC. Team composition, physical space, and hours of operation vary between organisations [13,15]. Evidence suggests that F-SDEC models can reduce ED waiting times and unplanned admissions [16,17,18].

While the intended value of F-SDEC is widely recognised [19], there remains a gap between the model’s theoretical promise and how it is implemented under routine conditions. This is particularly important in systems under operational pressure, with workforce instability, and with a fragmented information infrastructure [16,20].

### Implementing F-SDEC: the need for embedded evidence

Existing evidence indicates that, while a F-SDEC has a strong rationale, but it is challenging to implement in practice. Studies repeatedly identify barriers, including difficulties with early identification and referral, workforce shortages, and unstable staffing, that hinder the delivery of CGA and limit access to rapid diagnostics and transport [16,21,22]. Additional challenges include variable integration with community services [16,22], and the repurposing of HCPs and space during high operational pressure [23,24]. Given F-SDEC’s reliance on coordinated MDTs, the lack of a single senior decision-maker can also hinder preventive planning and follow-up [25,26], while the additional workload created by new pathways may increase pressure on existing teams [18]. Without robust integration with the community and secondary care, F-SDEC services risk creating fragmented care with quality, safety, and cost implications [26].

In this sense, F-SDEC also depends on how well the wider system is integrated: delivering a prevention-oriented pathway requires operational alignment and collaboration within the hospital and extends into primary, community, and social care services. However, digital, organisational, and workforce constraints often limit integration in practice, with direct implications for whether F-SDEC can function as intended [27,28].

Importantly, despite this established picture of the barriers that exist, there is less embedded evidence on how these barriers are experienced, negotiated, and adapted in situ, or on how the implementation climate shapes service functioning and outcomes. Implementation science emphasises that accurate service appraisal requires understanding how interventions are taken up, enacted with fidelity, adapted in context, and sustained [29,30]. However, measuring implementation success often relies on poor metrics and retrospective self-report, which poorly align with actual practice and may not reflect practitioners’ real-world decision-making [31]. This strengthens the case for naturalistic, embedded approaches that can illuminate how contextual constraints shape organisational behaviour and, in turn, the feasibility of preventive models in pressured acute systems [32].

### Study settings and contribution

Torbay and South Devon NHS Foundation Trust (TSDFT) serves a peripheral coastal region with around a quarter of residents aged 65 or over [33]. Like other coastal regions, it faces heightened health needs alongside workforce shortages and fragmented digital systems [34]. In such settings, where capacity and infrastructure constraints are concentrated, the gap between the theoretical promise of F-SDEC and its practical implementation may be particularly pronounced. Reflecting this, Knight et al. [16] have called for studies examining barriers to implementation in hospitals struggling to provide frailty services to support system-wide learning and change.

Collectively, these dynamics highlight the need for implementation-focused research examining how F-SDEC services are experienced and constrained in practice. While previous research has catalogued barriers and facilitators, this study demonstrates how pressures across triage, workforce capacity, and information infrastructure intersect to shape day-to-day service viability. By developing this account in a challenging setting, the paper offers transferable lessons for similar systems seeking to address compounding barriers early and expand frailty-focused urgent care.

## Aims and Objectives

This paper explores how HCPs experience and navigates the implementation of an F-SDEC service in a coastal NHS hospital in Southwest England. To this end, it seeks to:

1. Examine how frailty triage and referral operate in practice, and how staff perceive their usefulness, limitations, and risks.
2. Identify key facilitators and barriers related to staffing, skill mix, and resource allocation within F-SDEC.
3. Explore the educational and cultural changes required to support holistic, integrated frailty care across hospital teams and community interfaces.

## Methods

### Approach

This research adapted the participatory Researcher-in-Residence (RiR) methodology, which values coproduced knowledge, naturalistic observation, and reflexive learning cycles [35,36]. Here, it was used to support relational access, contextual understanding, sensitivity to the everyday realities of service delivery, and iterative feedback within a coastal healthcare organisation. Guided by a pragmatic lens [37], what is presented below should not be understood as a set of objective facts but as in-practice experiences emerging from the interaction between empirical observation, reflexive engagement, and knowledge exchange.

### Context

The project took place at the TSDFT main hospital site. TSDFT was redesigning its older people’s care pathways to address winter pressures in the ED, organisational stress, and financial deficit, and to enable an integrated approach to frailty care that reflected the needs and preferences of its older demographic. Prior efforts to manage ED pressures through an ED In-reach model and bespoke frailty care in AMU had limited success. At the time of the pilot, TSDFT also had a recently established Frailty Hospital at Home service, live for approximately three months, which was beginning to work in tandem with F-SDEC as a step-down option and source of additional support for patients discharged home with ongoing monitoring or care needs. In line with national guidance and supported by evidence [16,17,18], TSDFT selected F-SDEC as a high-impact intervention [19]. The pilot was implemented in the context of workforce gaps (particularly allied health professional capacity) and fragmented digital infrastructure, which influenced how the model was staffed and operationalised.

### Intervention

Trust managers and acute frailty practitioners collaborated to develop and pilot the F-SDEC service in January 2024, which concluded in June 2024 (approximately 6 months). The F-SDEC service was designed as a non-bedded unit, located adjacent to the hospital’s discharge lounge, open weekdays, from 8:00 AM to 6:00 PM. The service initially operated with a capacity of three patients at a time, aiming to grow to five by the end of the pilot. Referrals came primarily from the ED (and occasionally AMU) and were screened by the frailty coordinator, with eligibility confirmed by the geriatrician in situ.

Trust executives envisioned F-SDEC as a mechanism to “decompress” the ED. It aimed to provide a dedicated pathway for patients requiring CGA, diagnostics when required, holistic care planning, and same-day discharge. Staffing typically included a geriatrician, one to two resident doctors, a physician assistant, a frailty coordinator, and nursing support. The team worked closely with the Joint Emergency Team (JET) and a newly established virtual ward, partly to mitigate vacancies in allied health professional roles. The pilot closed following a decision not to continue the model beyond the pilot period.

The RiR, embedded within the Frailty and Health Care for Older People teams under an honorary contract, was invited by the hospital’s strategic management to observe and analyse the pilot’s implementation. The study was approved by the Health Research Authority (IRAS ID: 322539) and formed part of a larger project.

### Sampling

Strategic management connected the RiR with the ward and team manager of the F-SDEC implementation site. Participants were selected via snowball sampling, leveraging connections made during RiR’s prior relationship-building. The sample included three of the four consultant geriatricians involved in running the F-SDEC, resident doctors, a Frailty Advanced Care Practitioner, and those helping facilitate the F-SDEC initiative (e.g., key strategists, team leaders, and managers) (Table 1). ED and AMU frontline staff were not included due to feasibility constraints, meaning the study focuses on perspectives of those most directly involved in F-SDEC design and delivery.

**Table 1:** Interview Participant Information.


INTERVIEW PARTICIPANT INFORMATION	

ROLE	PARTICIPANT CODE

Consultant Geriatricians	HCP2; HCP4; HCP5

Frailty Advanced Care Practitioner	HCP1

Resident Doctor	HCP3

Head of the Frailty Virtual Ward	HCP7

Frailty Team Lead	HCP6


### Data collection

Data collection followed a sequential design: early observation, followed by interviews. First, over 16 months of prior relationship-building with the frailty team, the RiR conducted around 40 hours of observational engagement with the frailty team, documented through reflexive field notes. These engagements informed the interview guide by sensitising the researcher to key areas impacting potential implementation, including variation in frailty skills, confidence, and ownership across teams, as well as issues in triage and referral processes, information sharing, and HCP burnout.

Second, seven semi-structured interviews (lasting 30 to 80 minutes) were conducted in April 2024, towards the end of the SDEC pilot, to capture HCPs’ experiences as the service was being operationalised. The interview schedule covered participants’ roles within the frailty system, their experience of the F-SDEC model in day-to-day practice, and their aspirations for the future of frailty care. Specific points of reflection included MDT relationships, determinants of a successful day, triage and patient selection processes, IT and coordination, and where a future F-SDEC service should be positioned along a care pathway. All interviews were conducted, recorded, and transcribed by the embedded RiR.

### Data analysis

All field notes were initially imported into NVivo. Analysis followed a framework-informed, reflexive thematic approach [38]. The RiR re-familiarised themselves with the data through repeated reading of the transcripts, field notes, and relevant literature. Early analytical themes were generated as sentences that captured observations, tensions, or patterns within the data, rather than as fixed categories.

These themes were cross-examined through the transcripts, supported by multiple instances of evidence. As further evidence was collated, themes were continuously defined and developed in complexity. Themes emerged inductively from transcripts and field notes, informed by interview topics and grounded in HCPs accounts of practice. This process resulted in several key themes; by incorporating interview evidence under each, it captured recurring patterns in how F-SDEC was experienced and implemented in practice.

Finally, after the pilot concluded, findings were presented to participants in a feedback session (June 2024) to support collective reflection and validation and to refine the interpretation of the data. Stakeholders broadly agreed with the study’s findings and noted how rapid service change had led to staff fatigue. These reflections informed the interpretation and analysis presented in this paper. Rigour was supported through triangulation across interviews and observational field notes, and a feedback session to sense-check interpretations.

### Co-production and reflexive learning processes

Although it is not possible to attribute the closure of the F-SDEC pilot to this research, the findings aligned with stakeholder reflections on the service’s limitations and contributed to an understanding of service sustainability under current conditions. In subsequent informal feedback, clinical HCPs indicated that the organisation was revisiting the F-SDEC concept following the implementation of an electronic patient record, suggesting that the study may have contributed to longer-term organisational learning rather than immediate service redesign.

## Results

### The F-SDEC offer

HCP accounts consistently framed the F-SDEC pilot as an attempt to offer holistic, preventive frailty care within an otherwise reactive acute system struggling to manage winter pressures in the ED. Many expected the unit to create protected time for comprehensive assessment and planning.

‘We’ve got to sort out the front door [ED]… everything is reactive, everything is about getting prepared for winter… Winter happens every year… Crisis, crisis. Crisis.’ (HCP7)

‘We were holistically and comprehensively assessing our patients to try and prevent them from coming back in… that’s probably about, I don’t know, 400 patient bed days we could save’ (HCP4)

The model was seen as enabling slower, more investigative work for frail patients who might otherwise have been quickly admitted and discharged without a comprehensive preventive care plan (HCP3). HCPs positioned F-SDEC not only as a quality improvement intervention, but also as an organisational strategy to intercept avoidable admissions and reduce bed pressures. The proactive, investigative style of F-SDEC care was a means of both pulling demand from an overcrowded ED and saving hundreds of acute bed days. This was seen as an attractive goal. However, HCPs identified several factors that limited their ability to implement F-SDEC effectively. These constraints shaped how the service functioned in practice and are explored in the sections below.

### Triage

#### IT services and lack of EPR

Across interviews, triage emerged as a central implementation barrier, where clinical and operational pressures intersected with competing expectations about F-SDEC’s purpose. HCPs leading the F-SDEC service found it very difficult to identify the right patients. Triaging patients in the ED was time-consuming due to various factors, including fragmented IT systems and non-interoperable systems, resulting in incomplete and sometimes inaccurate information across the ED, ambulance, and frailty teams.

‘Computer systems in this hospital are not the best at joining up data about patients because different departments use different systems… things like clinical frailty score, although A&E must fill it in, are very likely to be inaccurate.’ (HCP2)

Due to data discrepancies, including incomplete Clinical Frailty Scales (CFS), the teams relied on screening multiple patient lists and attending the ED for in-person decision-making, which contributed to delays. As a result, triage expanded from a bounded administrative task into a form of manual and relational labour, reliant on clinician presence and informal negotiation rather than system-level visibility.

#### Difficulty locating correct patients in ED

Eligibility criteria also proved difficult to apply because of contextual factors (e.g., HCP availability, space, and the potential need to admit overnight). HCPs described ‘ideal’ candidates for F-SDEC as having a CFS of 5 or 6, being mobile, safe at home, and supported by a package of care. They felt this level of assessment was unrealistic to expect from ED teams under pressure.

‘The list [eligibility criteria] keeps going and so we’ve never got to a point where we felt we could ask another team to pick our patients… We’ve tried to expand our criteria for who we’ll take out of the emergency department… there’s a lot of pressure, like just make sure you find 5 patients today, and you go, well, that’s not really the point…’ (HCP2)

As a result, ED teams could not reliably identify suitable patients independently, and F-SDEC clinicians often had to screen multiple lists and undertake additional in-person assessments in the ED before patients could be transferred. This extended triage from a bounded administrative task into time-intensive clinical labour, contributing to delays and, in some instances, prolonged waiting. Staff described occasions where patients deteriorated, necessitating admission. This generated tensions between the desire to protect a prevention-oriented model of care and the operational expectation that F-SDEC should function as a mechanism to relieve ED pressure.

‘There are patients down there [F-SDEC] waiting for quite some time…. The hope was that we would be able to get them home, but they’ve now gone into a fast heart rhythm…’ (HCP4)

Inconsistencies in decision-making between different consultants and challenges in managing patients’ expectations further complicated triage processes. Some consultants supported F-SDEC staff in identifying potentially eligible patients in ambulances or at AMUs; others did not. Patients also struggled when hospital-based assessments contradicted prior advice from trusted primary clinicians.

Taken together, data fragmentation, changing eligibility criteria, inconsistent decision-making, and varying understandings of F-SDEC’s purpose across hospital hierarchies interacted to create a triage experience that was unpredictable and difficult to routinise. Rather than enabling the timely identification of suitable patients for preventive frailty care, the in-practice processes constrained F-SDEC’s ability to function as a predictable, prevention-oriented alternative to admission. In the next section, we outline a triage solution presented by HCPs, with a focus on integrated community services and earlier intervention.

#### A community facing SDEC

With ongoing challenges in ED triage, key members of the F-SDEC team increasingly reframed where and how it could generate value. Rather than drawing patients from the ED, where many were perceived as too unwell to benefit fully from a same-day model, they were increasingly convinced they should reorient towards accepting referrals from the community. They believed that this earlier intervention via general practitioner (GP) referrals would better align with the service’s preventive intent.

‘Basically, the team have always said the people we want are the people in the community. We want to stop them rocking up at ED to begin with.’ (HCP6)

This proposed shift aimed to resolve the tension between prevention-oriented care and ED-led triage. In practice, however, this proved difficult to operationalise due to limited reciprocal access and data sharing between primary care, community services, and the hospital. Utilising AMU as an F-SDEC triage hub was also an attractive idea. However, concerns about acute medical workload and consultant shortages, reflected in high locum use, made implementation difficult.

HCPs’ capacity within F-SDEC itself would also need to be addressed to handle community-based referrals:

‘Ideally, we’d have some booked slots and some ad hoc slots that we could use… we’d have 3 admissions, 3 booked slots a day… [However], I think we would need the backfill for all the positions we have.’ (HCP2)

In this sense, while the aspiration to shift towards community-based referrals reflected a perceived misalignment between ED-led triage and preventive care, HCPs also recognised that the same digital, relational, and workforce constraints restricted the viability of alternative referral routes. Despite this, HCPs maintained a strong appetite for a proactive, holistic frailty care model. Looking ahead, they saw that preventive referrals supported by greater data integration were central to the long-term value of F-SDEC.

### HCP capacity

#### Capacity within the SDEC team

Staffing capacity within the F-SDEC team emerged as a key factor shaping the consistency and reliability of the F-SDEC offer. As noted above, the inability to backfill HCPs constrained the possibility of delivering a community-facing F-SDEC service. Required roles included staff who could undertake a CGA and a physiotherapy assessment. Given the need for testing and observation (e.g., X-rays and blood tests), nursing staff relied on support from healthcare assistants.

Reflecting broader recruitment issues within this coastal trust (allied health professional roles were particularly affected by vacancies), these roles were often unfilled or understaffed. As a result, absences due to annual leave or illness had a disproportionate impact. Participants set a target of 10 patients per day, but staffing levels often limited throughput to around 5, and sometimes as low as 2. HCPs also worried about the consistency of assessments when specialist physiotherapy or occupational therapy input was unavailable and had to be covered by non-specialists (e.g., trainee ACPs) or when only partial geriatric assessment could be completed.

HCPs also described the physical and emotional strain of maintaining the service with limited personnel. Regular late finishes, missed breaks, and unsustainable workloads were common.

‘She works from 8 to 4, so today is the only day that she is leaving at 4. So, the number of days that she has left the work on time over four months would be less than five. Sometimes we miss our lunch, or like it’s usually very late lunch… everyone is trying their best to keep this SDEC going.’ (HCP3)

‘The current way they’re [the SDEC team] having to work is unsustainable… I think that’s why they’re all sick, like joking aside. Yes, they’ve clearly all caught the same thing this week, but… we’ve got one person in each role.’ (HCP7)

In reflection, these experiences suggest that HCP shortages were not simply a capacity constraint but also a source of day-to-day variability that limited the reliability of the F-SDEC service and its ability to deliver care as intended. For HCPs, this variability appeared to contribute to fatigue, emotional strain, and an increased risk of burnout.

#### Capacity in other parts of the system

The availability and responsiveness of other hospital services also shaped capacity within F-SDEC. HCPs highlighted how delays in diagnostics, health checks, and medication access reduced the F-SDEC teams’ capacity to support eligible patients’ same-day discharge. Patients requiring an X-ray or CT scan would require a porter to transfer them. Participants described waiting hours for a member of the orthopaedic or trauma team to respond to calls. Even accessing simple medication like paracetamol involved a trip to AMU or the discharge lounge.

‘I was trying to chase the orthopaedic team since like 11:30… at 3:00 PM my consultant called the trauma coordinator… by the time they came and assessed the patient, it was 16:00’ (HCP3)

These logistical problems may, in part, have reflected the F-SDEC team’s newness, with other departments unclear about the unit’s remit and urgency. This indicates a need for clearer communication and engagement with other departments regarding F-SDEC’s remit and time-sensitivity. Regardless of cause, these delays had direct implications for a time-limited, same-day model of care.

Taken together, delays in diagnostics, portering, and medication access interacted with difficulties in consistent triage processes in the ED and HCP capacity. These system-level frictions compounded already unstable workflows, further constraining F-SDEC’s ability to deliver timely, prevention-oriented care. In this sense, HCPs experienced barriers at multiple points along the pathway, from triage, to internal capacity, to the timely response of other teams required to complete the service’s intended purpose.

#### Cultural and educational barriers to frailty care

Alongside operational and capacity-related challenges, HCPs reflected on a broader cultural challenge: reshaping how frailty was recognised and responded to across the hospital. Although they recognised the significant impact of frailty on hospital demand, HCPs outlined the difficulties of reconciling the complexity of frailty, with its often less clear-cut pathways or treatment plans, with the fast-paced, diagnosis-driven care that dominates hospital practice. They also described how progress in frailty care can be incremental rather than immediately visible, alongside a tendency for some clinicians to prioritise biomedical issues over functional, psychosocial, and relational aspects of care.

‘There’s a bit of that ageism there… [frail patients] come in with very vague presentations. There’s no instant gratification… It’s more like, oh, you’ve had a fall, you were confused, and you’re still confused.’ (HCP5)

In this context, HCPs suggested that referrals were sometimes shaped by age-based assumptions rather than a shared understanding of frailty as a dynamic and multidimensional condition. This contributed to uncertainty about who should be referred to F-SDEC and when. Participants described examples where very old patients who were not particularly frail were referred, while others with acute conditions (e.g., pneumonia) were referred despite being unlikely to benefit from a same-day frailty pathway. HCPs emphasized that this did not reflect a lack of commitment to caring for older patients, but rather a lack of understanding about how to identify and manage frailty and of the relative roles of medical specialties and the F-SDEC service.

This framing suggests the need for improved frailty literacy, cross-disciplinary trust, shared responsibility, and greater confidence in other teams. Without this, HCPs felt prevention-oriented models such as F-SDEC would continue to struggle to operate as intended within acute care settings.

## Discussion

### Summary and interpretation of key findings

This study aimed to explore the implementation of an F-SDEC pilot through the experiential accounts of HCPs involved in day-to-day delivery. Findings reflect the mid-to-late phase of a time-limited pilot operating at relatively low and variable throughput. Accordingly, this should be read as a qualitative account of pilot delivery and adaptation under pressure rather than an evaluation of service effectiveness. HCPs regarded F-SDEC as a compelling model to reduce ED pressure and enable holistic assessment and discharge planning for frail older people.

Implementation was constrained by intertwining organisational and system factors, including time-intensive triage under operational pressure, fragmented IT and poor record interoperability, workforce capacity and skill-mix gaps required for CGA delivery, and variable understanding and ownership of frailty across hospital teams. A further finding was a tension between the F-SDECs’ preventive care and the operational expectation that it should relieve ED pressures. This tension, HCPs suggested, led to “purpose drift” as the pilot progressed.

### The limits of ED-led triage in fragmented systems

HCPs consistently described triage as a highly complex, time-intensive, and uncertain process. Decisions were shaped by the variable availability and reliability of frailty indicators (e.g., CFS) across fragmented IT systems, the quality of information gathered from patients, and the ED’s operational environment [37]. Together, these factors led to inefficient manual screening processes, resulting in reduced throughput and delayed assessments. These findings align with the broader literature, which highlights how poor interoperability of data across healthcare sectors, such as between GPs, VCSE organisations, hospital teams [39,40], community services [41], and other F-SDEC services [13,42], impedes timely frailty identification and appropriate care planning. Indeed, many SDEC pilots appear to exclude frail patients due to difficulties in identification and uncertainty about service suitability [16,43].

Importantly, our study highlighted that HCPs experienced triage problems as more than a technical issue. In ED, HCPs repeatedly attempted to adapt eligibility criteria to ensure they found the right patients yet reported that many patients identified in the ED were perceived to be too unwell. This became a mechanism through which expectations of F-SDEC as an ED pressure-relief mechanism clashed with the team’s desire to carefully select the right patients as to ensure they can offer prevention-oriented care.

However, hospital managers continued to expect F-SDEC to provide ED relief. For HCPs, without access to relevant shared care records, clear eligibility criteria, and sufficient HCPs to absorb community referrals, this was highly challenging. As a result, overall patient numbers remained low, and the pilot struggled to evidence value against prevailing expectations. Improving digital integration is therefore central to F-SDEC’s success. National policies indicate that shared care records can support timely frailty assessment, facilitate MDT collaboration, and thus, enable safe discharge planning [44].

### Workforce capacity and service fragility in peripheral settings

Workforce constraints were not an isolated barrier but a compounding condition shaping service reliability. Our study identified a lack of consistency and the necessary skill mix, particularly adequate therapy and nursing support, alongside limited backfill. This resulted in day-to-day variability in triage considerations, the number of patients who could be seen, and which components of assessment could realistically be delivered. This variation in a planned model’s deliverability can undermine cross-sector trust in its value; teams, such as ED, may be less willing to refer [45]. Additionally, instability can erode the piloting team’s morale, as HCPs struggle to achieve the service’s key purpose. In this study, HCPs were already struggling to identify suitable patients by repeatedly adapting their eligibility to ensure patients could be found. Add to this an inability to provide the necessary support for when an eligible patient is found, and HCPs described fatigue, stress, exhaustion, and burnout [46].

Understanding the specific interactions between workforce restraint, cross-sector confidence, and team morale may be particularly relevant for other coastal and peripheral trusts. Multiple sources highlight national workforce shortages, specifically a lack of geriatric specialists, frailty, and social care practitioners [15,16,42]. However, in coastal and peripheral trusts, recruitment and retention challenges compound with the complex health and care needs of an ageing population, adding further implementation risk [43,47]. Absences and vacancies can rapidly destabilise new services and compound wider operational delays (e.g., diagnostics and portering), increasing pressure on staff and reducing throughput, thereby reinforcing ED pressures.

Taken together, the data suggest that early pilots may require realistic expectations about capacity and explicit investment in protected staffing and backfill if services are to demonstrate value and mature. Where ED-based triage depends on specialist input, F-SDEC is also likely to require careful integration across the wider pathway to achieve its intended value.

### Culture change and training

Alongside operational constraints, HCPs noted that effective F-SDEC implementation required cultural change, with responsibility for frailty care shared across ED teams, acute physicians, geriatrics, and community services. However, there was variation in frailty literacy, confidence, and perceived ownership across services and specialties. This variation across teams contributed to inconsistent referrals, uncertainty about eligibility, and concerns that age-based assumptions were sometimes being conflated with frailty.

These findings align with guidance from the World Health Organisation, the British Geriatric Society (BGS), and the NHS, which stress that meeting the complex service needs of frail patients requires shared approaches to frailty identification and management across settings [39,40,48]. They also echo qualitative studies suggesting that simply adopting new frailty care policies is insufficient; clinicians throughout the care continuum must regard frailty as a key organising principle for care if new models are to function as intended [16].

### Contribution to existing literature

The barriers identified in this study, from triage to care cultures, are described in prior literature on F-SDEC or acute frailty services. The unique contribution of this paper, therefore, is to integrate these strands into a grounded account, exploring how they were experienced concurrently during F-SDEC implementation, particularly within a geographically peripheral coastal trust. In this sense, the paper unearths implementation dynamics that are less visible in prior literature. For example, it shows how inconsistent team capacity and skills mix undermined confidence, potentially shaping referral behaviour and team morale. By articulating these dynamics, the study offers practice-relevant insights for teams attempting to implement F-SDEC models in resource-constrained and peripheral settings.

### Implications for clinicians and system leaders

For acute frailty clinicians, the findings validate that difficulties in triage, staffing, and cross-sector understanding of frailty are often interdependent and can push prevention-oriented models toward reactive flow work under ED performance pressures and digital fragmentation.

For system leaders and policy makers, the study suggests that achieving the intended benefits of F-SDEC requires: (i) interoperable information flows to support timely frailty identification and shared care planning; (ii) protected staffing and backfill to sustain core MDT functions required for a CGA; (iii) explicit agreement with ED and acute medicine regarding service purpose and referral logic; and (iv) investment in shared frailty education and role clarity across the pathway. Without these enabling conditions, particularly in peripheral and resource-constrained settings, services may struggle to establish a stable offer or to demonstrate value early enough to survive pilot phases.

## Conclusions

This study indicates that while F-SDEC services align with strong national policy ambitions [17], their implementation is highly contingent on local digital infrastructure, workforce capacity, and a shared understanding of the service’s purpose. Consequently, such top-down policies encounter significant implementation barriers, particularly in digitally immature and geographically rural or coastal environments.

By examining F-SDEC implementation in this context, this study provides a grounded account of how interdependent barriers can limit a service’s ability to stabilise, mature, and demonstrate value within a pilot timeframe. In this case, difficulties in triage, staffing, and cross-team alignment persisted over time, constraining throughput and confidence in the model.

Taken together, these findings highlight a persistent gap between national ambitions for frailty care and local readiness to deliver them in practice. They suggest that successful F-SDEC implementation depends on treating frailty care as a whole-system endeavour, requiring alignment across acute, community, and information infrastructures rather than relying solely on ED-led identification. Future evaluations should therefore attend not only to performance metrics, but also to the conditions that enable services to function as intended during early implementation.

## Limitations of this Study

This was a single-site qualitative study conducted in a coastal NHS hospital, which limits the transferability of the findings. Although the data are rich and grounded in the lived experiences of HCPs, caution should be exercised in generalising the results to other regions or health systems. Nevertheless, the themes identified, such as digital infrastructure challenges, workforce constraints, and fragmented cross-sector collaboration, are likely to be relevant across multiple settings.

The RiR model facilitated deep contextual understanding but may also have introduced interpretative bias due to the researcher’s dual role as both observer and participant. This risk was mitigated through triangulation with participants and critical review of emerging themes, though it cannot be entirely excluded. A further limitation concerns the limited opportunity to fully implement iterative service-design feedback loops, which are often a strength of RiR approaches [36]. Due to the short duration and timing of the pilot, the study focused primarily on observation, interpretation, and shared reflection rather than on continuous, active formative feedback to shape service redesign. Future implementation studies could more fully explore the potential of RiR models to support real-time learning and adaptive change during pilot delivery.

Finally, the study was conducted during the early implementation phase of the F-SDEC model, limiting insights into longer-term outcomes, sustainability, and broader system-level effects. While the study foregrounded staff perspectives, the absence of patient and caregiver voices restricts the breadth of evaluation. The study also did not employ a structured implementation science framework (e.g., CFIR or RE-AIM), which could have enabled a more systematic analysis of multilevel implementation dynamics. Future research would benefit from applying such frameworks, incorporating more diverse stakeholder perspectives, and adopting longitudinal, multi-site approaches to understand better the conditions under which F-SDEC can be successfully embedded and sustained.
